# Robson classification of caesarean births: implications for reducing caesarean section rate in a private tertiary hospital in Nigeria

**DOI:** 10.1186/s12884-023-05557-x

**Published:** 2023-04-12

**Authors:** Adebayo Adekunle Akadri, John Osaigbovo Imaralu, Omotayo Felicia Salami, Chimaobi Chukwuemeka Nwankpa, Akinmade Adekunle Adepoju

**Affiliations:** 1 Department of Obstetrics and Gynaecology, Babcock University, Ilishan-Remo, Nigeria; 2 Department of Surgery, Babcock University, Ilishan-Remo, Nigeria; 3 Department of Community Medicine, Babcock University, Ilishan-Remo, Nigeria

**Keywords:** Births, Caesarean section, Delivery, Rate, Robson, Nigeria

## Abstract

**Background:**

Caesarean section (CS) is a potentially lifesaving obstetric procedure. However, there are concerns about the rising CS rate in many countries of the world including Nigeria. The Ten-Group Robson classification system is presently recommended as an effective monitoring tool for comparing CS rates and identifying target groups for intervention aimed at reducing the rates. The aim of this study was to evaluate the cesarean section rate and the groups with the highest risk of CS at the obstetric unit of Babcock University Teaching Hospital (BUTH), using the Robson classification system.

**Methods:**

A cross-sectional study involving 447 women who gave birth at the obstetric unit of BUTH between August 2020 and February 2022. Relevant information was retrieved from the delivery records of the study participants. Data were analyzed using the IBM-SPSS Statistics for Windows version 23.0 (IBM Corp., Armonk, NY, USA).

**Results:**

The overall CS rate was 51.2%. Multiparous women with previous CS, single, cephalic, term (group 5); nulliparous women, single cephalic, term, with induced labour or pre-labour CS (group 2); women with preterm single cephalic, term (group 10); and single cephalic term multiparous women in spontaneous labour (group 3) were the largest contributors to CS rate accounting for 34.5%, 14.0%, 12.6%, and 10.0% respectively. The commonest indication for CS was previous CS (87; 38.0%), followed by poor progress in labour (24; 10.5%).

**Conclusions:**

The CS rate in BUTH is high and Robson groups 5, 2 10 and 3 were the major contributors to this high rate. Interventions directed at reducing the first CS by improving management of spontaneous and induced labours; and strengthening clinical practice around encouraging vaginal birth after CS will have the most significant effect on reducing CS rate.

## Background

Caesarean section (CS) is a potentially lifesaving obstetric procedure often performed when it is determined that vaginal delivery could be harmful to either mother or the baby [1, 2]. It essentially involves delivering a foetus by making an incision on the mother’s abdomen and the uterus after the age of viability [3].

A survey of 150 countries reported the average worldwide CS rate to be 18.6%, with range of 6–27.2% in the least and most developed countries respectively [4]. Among the regions of the world, Africa has the lowest population level CS rate (7.3%) while Latin America and Caribbean regions have the highest (40.5%) [4]. Caesarean section rate also varies from one health facility to the other within the same country [5]. In Nigeria, facility level CS rates of 27.6% and 32.9% were reported in Enugu and Sagamu respectively [3, 5].

There is a global concern about the rising CS rate and this is particularly dramatic in many middle- and high income countries, but at a lower degree in low income countries [4, 6]. The factors responsible for the rising CS rates are still subject to debate. Factors such as fear of litigation, changing maternal characteristics, use of electronic foetal monitoring, and changing professional practice styles have been implicated by some authors [3, 4, 7]. Some other researchers have postulated that socio-cultural and economic factors as seen in many cases of non-medically indicated CSs also propel the rise in CS rate [4, 8]. There is evidence that suggests that CS rates are higher in private health facilities compared to public facilities [9]. It is believed that economic factors and maternal preferences are the most significant reason why the CS rate differs between public and private health facilities [9]. Private health facilities are also more than twice as likely to have undefined indications for CS compared to public health facilities [10].

The World Health Organization has suggested that a population-based CS rate higher than 10% is not associated with any additional benefit for mother and baby [11, 12]. Reports from surveys indicate that CS rates in many obstetric units in Nigeria are higher than the WHO threshold, and have been rising over the past few decades [3, 5]. Although CS is a safe procedure when done by trained medical personnel, the global increasing CS rate is a cause for concern. This is because CS may be associated with some maternal and neonatal complications affecting the index or future pregnancies [1, 13]. Such complications include increased need for blood transfusion, postpartum infections, retained placenta, stillbirths and postpartum haemorrhage. Others are placenta previa, morbidly adherent placenta, and uterine rupture with possibility of peripartum hysterectomy thus jeopardizing future reproductive life [1]. Compared with vaginal delivery, the procedure is also associated with increased health care costs [4, 13]. While it is desirable to reduce the rate of CS in Nigerian obstetric units, it should be borne in mind that ensuring access to medically justifiable CS is an essential strategy to reduce maternal and perinatal morbidity and mortality [13]. Hence, it is very important to study the characteristics of women receiving the procedure and whether the procedure is being done for justifiable reasons [14]. It is also important to examine the reasons for the CS trend in different health facilities and population of women [15]. In order to achieve this, there is need for adoption and consistent use of an internationally accepted classification system that has been proven to enhance the analysis and comparison of CS rates at various settings in a consistent manner and transform this data into useful information [14, 16]. The Ten-Group Robson classification system is presently recommended by the WHO and International Federation of Gynecology and Obstetrics (FIGO) as an effective monitoring tool for comparing CS rates within various obstetric units over time as well as between them [12, 17]. This system uses obstetric characteristics like parity, gestational age, previous CS, labour onset (spontaneous or induced), presentation and number of foetuses (singleton or multiple) to classify women into ten groups [18] There is some evidence to suggest that the use of the Robson classification system for auditing CS in health care facilities may result in reduced CS rates [11].

There is sparse data on the use of the Robson’s classification for CS in Nigeria, although reports from many tertiary health facilities in the country suggest that the CS rate is higher than the WHO recommendation. The aim of this study is to evaluate the caesarean rates and the groups with the highest risk of CS at the obstetric unit of Babcock University Teaching Hospital, using the Robson classification system.

## Methods

### Study design

This cross-sectional study was conducted in the obstetric and anaesthetic care units of Babcock University Teaching Hospital (BUTH), Ogun State Nigeria.

### Study setting

BUTH is a faith-based tertiary health facility which provides care for patients from the university community, and from adjoining towns and cities in Ogun and Lagos states in Nigeria. There are 24 Obstetric beds in the health facility and approximately 350 births are attended annually. Obstetric care is provided by five Consultant Obstetricians, complemented by resident doctors, nurses and midwives. The health facility has one fully functional obstetrics theatre suite manned by Consultant Anaesthetists. BUTH also provides paediatric and blood transfusion services. The target population were women who gave birth at the obstetric unit of BUTH.

### Sample size

All women who gave birth at BUTH between August 2020 and February 2022 were included in the study.

### Data collection

The case files of all the women included for the study were retrieved and relevant information extracted. A data capture sheet specifically designed for this study was used to extract information on the maternal characteristics including age, parity, gestational age, number of fetuses, foetal presentation, the clinical indications for surgery, induction of labour (Yes or No), type of CS (elective or emergency), fetal outcome (live or still birth), birth weight and Apgar scores of babies. The CSs performed during the study period were classified using the Robson ten group classification system with subdivisions [19] (Table 1). This classification is based on six major obstetric variables- onset of labour (spontaneous or induced), parity, gestational age (weeks), fetal presentation, number of fetuses, and previous caesarean delivery [18]. The Robson group for each CS was recorded on the data capture sheet.

**Table 1:** Robson classification with subdivisions

Robson Group	Clinical characteristics
1	Nulliparous, single cephalic, ≥ 37 weeks, spontaneous labor
2 2a 2b	Nulliparous, single cephalic, ≥ 37 weeks, induced labour or CS before labour Labour induced Pre-labour CS
3	Multiparous without previous CS, single, cephalic, ≥ 37 weeks, spontaneous labour
4 4a 4b	Multiparous without previous CS, single, cephalic, ≥ 37 weeks, induced labour or CS before labour Labour induced Pre-labour CS
5 5.1 5.2	Multiparous with previous CS, single, cephalic, ≥ 37 weeks With one previous CS With two or more CSs
6	All nulliparous breeches
7	All multiparous breeches (including previous CS)
8	All multiple pregnancies (including previous CS)
9	All transverse or oblique lies (including previous CS)
10	All preterm single cephalic, < 37 weeks (including previous CS)

### Data analysis

Data were analyzed using the IBM-SPSS Statistics for Windows version 23.0 (IBM Corp., Armonk, NY, USA). Analysis of the CSs in each Robson group was conducted to determine the contribution of each group to the total CSs (the number of CS in index group divided by the total number of women undergoing CS) and group contribution to the overall CS rate (the number of CS in index group divided by the total number of women giving birth) [14]. The normality of distribution of continuous variables was tested by Kolmogorov-Smirnov test. Continuous variables like maternal age, gestational age at delivery, birth weight, and Apgar scores were summarized using median (Q1-Q3). Categorical variables were summarized using frequencies and percentages.

## Results

A total of 447 women gave birth during the study period, and all of them were included in the study. The median age of study participants was 31years (Q1-Q3: 27–34 years). Two hundred and sixty women (58.2%) were aged 30 years and above. One hundred and fifty five women (34.7%) were nulliparous while 150 (33.6%) had parity of 2 and above. The median parity was 1 (Q1-Q3: 0–2). Majority (332;74.3%) gave birth at term. The median gestational age at delivery was 38 weeks (Q1-Q3: 37–39 weeks). Four hundred and twenty seven women (95.5%) had singleton pregnancies and cephalic presentation. Majority of the babies (290; 64.9%) had birth weights between 2.5 and 3.9 kg. The median birth weight was 3.2 kg (Q1-Q3: 2.8-3.5 kg). Most of the babies (420; 94%) had fifth minute Apgar score of seven and above. Twenty six women (5.8%) had induction of labour, while 127 women (55.5%) had elective CSs (Table 2).

**Table 2:** Demographic and obstetric characteristics of women who gave birth at BUTH, Nigeria between August 2020 and February 2022

Variable	N	%
Maternal age (years)		
< 30	187	41.8
≥ 30	260	58.2
Parity		
0	155	34.7
1	142	31.8
≥ 2	150	33.6
Gestational age (weeks)		
< 37	85	19.0
37–40	332	74.3
> 40	30	6.7
Presentation		
Cephalic	427	95.5
Non Cephalic	20	4.5
Number of foetuses		
Singleton	427	95.5
Multiple	20	4.5
Newborn Birth weight(kg)		
< 2.5	65	14.5
2.5–3.9	290	64.9
≥ 4.0	92	20.6
Fifth minute Apgar Score		
< 7	27	94.0
≥ 7	420	6.0
Induction of labour		
Yes	26	5.8
No	421	94.2
Caesarean Section (n = 229)		
Elective	127	55.5
Emergency	102	45.5

Table 3 depicts the contribution of each of the Robson obstetric groups to the overall CS rates. Out of the total number of 447 deliveries during the study period, 229 women had CSs giving an overall CS rate of 51.2%. Robson group 5 had the largest input to total CS (34.5%) and the largest contribution to the overall CS rate (17.7%) while Robson group 6 had the smallest input to total CS (2.2%) and smallest contribution to overall CS rate (1.1%). All the women in groups 6 and 9 had CSs i.e. group specific CS rate of 100%. Women in group 3 had the smallest group specific CS rate (16.9%). The commonest indication for CS was previous CS (87; 38.0%), followed by poor progress in labour (24; 10.5%) (Table 4).

**Table 3:** Contribution of Robson ten obstetric groups to the overall caesarean section rate

Robson Group	Number of women (n1)	Relative size of Robson group (%) n1/N1	Number of CS (n2)	Group specific CS (%) n2/n1	Group input to total CS (%) n2/N2	Group input to overall CS rate (%) n2/N1
1	86	19.2	21	24.4	9.2	4.7
2 2a 2b	38 19 19	8.5 4.3 4.3	32 13 19	84.2 68.4 100.0	14.0 5.7 8.3	7.2 2.9 4.3
3	136	30.4	23	16.9	10.0	5.1
4 4a 4b	18 7 11	4.0 1.6 2.5	15 4 11	83.3 57.1 100.0	6.6 1.8 4.8	3.3 0.9 2.4
5 5.1 5.2	81 66 15	18.1 14.8 3.4	79 64 15	97.5 97.0 100.0	34.5 27.9 6.6	17.7 14.3 3.3
6	5	1.1	5	100.0	2.2	1.1
7	9	2.0	8	88.9	3.5	1.8
8	20	4.5	11	55.0	4.8	2.5
9	6	1.3	6	100.0	2.6	1.3
10	49	11.0	29	59.2	12.6	6.5
Total	447	100.0	229	NA	100.0	51.2

CS = Caesarean Section; N1 = Total births (447); N2 = Total CS births (229); n1 = number of women in each Robson group; n2 = number of CS births in each Robson group; NA = not applicable

**Table 4:** Indications for caesarean section within each Robson group in women who gave birth at BUTH, Nigeria

INDICATIONS	1	2	3	4	5	6	7	8	9	10	N (%)
PCS	0	0	1	1	70	0	3	4	0	8	87(38.0)
PPL	4	5	10	2	1	0	0	0	0	2	24(10.5)
CPD/OL	8	7	3	3	0	0	0	0	0	0	21(9.2)
HDP	0	6	0	2	3	0	0	1	0	7	19(8.3)
FD	5	3	5	2	0	0	0	1	0	1	17(7.4)
MR	0	5	1	2	2	0	0	0	0	2	12(5.2)
BP	0	0	0	0	0	5	5	0	0	0	10(4.4)
M/AL	0	0	1	0	0	0	0	0	6	0	7(3.1)
MP	0	0	0	0	0	0	0	5	0	0	5(2.2)
PD/PT	1	3	0	0	1	0	0	0	0	0	5(2.2)
APH	0	0	1	0	1	0	0	0	0	2	4(1.7)
PTL	0	0	0	0	0	0	0	0	0	3	3(1.3)
ANRF	0	0	0	1	0	0	0	0	0	1	2(0.9)
OTHERS	3	3	1	2	1	0	0	0	0	3	13(5.7)

PCS (Previous caesarean section), PPL(Poor progress in labour), CPD/OL(Cephalopelvic disproportion/ Obstructed labour), HDP(Hypertensive disorders of pregnancy), FD(Foetal distress in labour), MR(Maternal request), BP(Breech presentation), M/AL(Other malpresentations / abnormal lie), MP(Multiple pregnancy), PD/PT(Postdate /post term pregnancy), APH(Antepartum hemorrhage), PTL(Preterm labour), ANRF(Antepartum non-reassuring foetus).

The distribution of foetal outcome for the various Robson groups is displayed in Table 5. The median gestational age at delivery was 34weeks (Q1-Q3: 30–36 weeks) for women in group 10 and 35 (33–36) weeks for those in group 8. The median birth weight was 2.2 kg (Q1-Q3: 1.7-2.6 kg) for groups 8 women and 2.3 kg (Q1-Q3: 1.4-3.0 kg) for group 10 women. The Median 5th minute Apgar score was 9 for all the groups.

**Table 5:** Distribution of foetal outcome within each Robson group in women who gave birth at BUTH, Nigeria

Robson Group	Gestational age (weeks)		Birth weight (kg)		5th minute Apgar Score	
	Median	Q1-Q3	Median	Q1-Q3	Median	Q1-Q3
1	39	38–40	3.2	3.0-3.5	9	9–9
2	39	38–41	3.5	3.1–3.8	9	9–9
3	39	38–40	3.3	3.0-3.5	9	9–9
4	38	37–39	3.5	2.7–3.7	9	9–9
5	38	37–38	3.2	2.9–3.5	9	9–9
6	37	32–39	2.9	1.6–3.9	9	9–9
7	38	36–38	3.1	2.7–3.6	9	8–9
8	35	33–36	2.2	1.7–2.6	9	9–9
9	38	37–40	3.2	2.4–3.4	9	8–9
10	34	30–36	2.3	1.4-3.0	9	6–9

## Discussion

The overall CS rate in this study was 51.2% with previous CS being the commonest indication. Robson groups 5, 2, 10, and 3 had the highest contribution to caesarean delivery in the health facility. The CS rate of 51.2% is considerably higher that the WHO recommended rate (19). Babah et al. reported CS rate of 51.3% from a study conducted in a public tertiary health facility in Lagos, Nigeria [20]. Other authors working in public tertiary health facilities in Nigeria have reported varying CS rates such as 21.4% in Abuja [21], and 42.4% in Bayelsa [22]. Studies have suggested that CS rates are often higher in private health facilities compared to public health facilities [14, 20]. There is a dearth of studies on CS in private health facilities in Nigeria. However, authors from private health facilities in Ethiopia and Italy reported CS rates of 34.5% and 59.2% respectively [9, 23]. It is noteworthy that in these settings, the CS rates have been reported to be lower in public health facilities (25.7% in Ethiopia and 30.4% in Italy) when compared to private facilities [9, 23]. Private health facilities are believed to allow more liberal use of CSs for social reasons or maternal requests [9, 10]. Another possible reason is fear of litigation which is more likely in clients of private health facilities than public health facilities [3]. Nevertheless, the relatively high CS rates in private facilities often suggests inappropriate use. Consistent use of the Robson classification in all health facilities will likely assist in identifying the obstetric population that disproportionately contribute to the high CS rate [14]. Monitoring these specific groups will allow interventions that may lead to reduction in non-medically indicated CSs.

According to WHO recommendation, the interpretation of the Robson classification report table requires assessments of three important domains: data quality, type of obstetric population, and CS rates [19]. One way of assessing data quality is to look at the CS rate in group 9. It is expected that this should be 100% as seen in this study. The ratio of ‘group 3 to 4’ is expected to be higher than ‘group 1 to 2’. This indicates good quality data based on WHO recommendations [19]. Regarding the population in this study, it should be noted that the combination of ‘groups 3 and 4’ (34.4%) is higher than combination of ‘groups 1 and 2’ (27.7%), indicating a slightly larger multiparous population. The 75th percentile parity of 2 suggests that majority of the women managed in BUTH were of relatively low parity. It has been suggested that Group 5 should account for about half of the total CS rate, and should be less than 10% in settings with low overall CS rate [19]. In this study, group 5 accounted for 18.1% which is less than half of the total CS rate of 51.2%. This size of group 5 was consistent with the high CS rate found in this study, and also suggests that there has been a high CS rate in the past years especially in Groups 1 and 2. The size of groups 6, 7 and 9 are not unusual whereas the relatively larger size of groups 8 and 10 are consistent with expected findings in a tertiary health facility.

Multiparous women with previous CS (Robson groups 5) and nulliparous women who had induction of labour or pre-labour CS (Robson group 2) together make up about a quarter of the obstetric population but contributed to almost half of the total CS. A similar pattern was reported in a study done in Brazil [24]. The fact that Group 5 alone contributed about a third of all CS may be a reflection of low rates of vaginal births after CS (VBAC). The standard practice in many obstetrics units is to consider VBAC in women with one previous CS who have no contraindications and recommend elective repeat CS for those that have more than one previous CS or have contraindications to VBAC [9, 25]. Majority (66; 81.5%) of women in group 5 had one previous CS (Robson group 5.1). Although this study did not enquire about the total number of women who planned or attempted VBAC, the high group specific CS rate (97.0%) in Robson group 5.1 suggests a very low VBAC rate. It is likely that many women with previous CS are not given enough support for the procedure due to fear of uterine rupture. One strategy that has been found to be successful in increasing VBAC rates is the setting up of dedicated VBAC clinics where women are adequately counselled and supported to make informed choices on the mode of birth for their next pregnancy [25]. Computer based decisions aids have also been employed to assist women in making decision on mode of delivery after a previous CS, with a resultant increase in VBAC rates [26].

Nulliparous women with single, cephalic presentation at term who had induction of labour or pre-labour CS (Robson group 2) had the second largest contribution to the total CSs in this study. The group specific CS rate for group 2 was also high (84.2%). High CS rates for this group have also been reported in other private facilities in Ethiopia (70.4%) and Bangladesh (99%) [14, 23]. The Robson guideline suggests that the CS rate in this group is usually around 20–35% [19]. Women in this group are arguably low risk women, hence the high CS rates indicates vast opportunities for interventions that may lead to reduction in CS rates. In this study, the size of groups 2a (induced labour) and 2b (pre-labour CS) are equal, however 68.4% of women planned for induction of labour (Group 2a) ended up having CS. Multiparous women without previous CS, single, cephalic, term who had induction of labour or pre-labour CS (Group 4) are also generally expected to have low CS rates. In this study the CS rate in this group (83.3%) was far higher than the rate suggested by the Robson guide (15%). However, 11 out of the 18 women (61%) in this group belonged to group 4b i.e. those who had pre-labour CS. Nevertheless, the high CS rate in group 4a (57.1%) also suggests poor success rate for induction or poor choice of women to induce. It is noteworthy that the labour induction rate in this study was 5.8%. A study carried out at another tertiary health facility in Ibadan, Southwest Nigeria reported a higher labour induction rate of 12.7% and lower failure rate of 36.5% (27). The poor success at induction of labour calls for review of induction of labour protocols in the health facility.

The third group that contributed most to the CS rate in this study was the preterm birth group (Robson group 10), contributing to 12.6% of the total CS performed and having a group specific CS rate of 59.2%. Robson group 10 was also the third largest contributor to CS rate in Brazil with a CS rate of 9.4% and group specific CS rate of 50.1% [28]. It is likely that the CSs carried out for this group of women are medically justified, possibly to improve perinatal outcomes.

The group specific CS rate for women with multiple pregnancies (group 8) was 55%, and this was lower than reports from Ethiopia (60%) and Iran (88%). The Robson guideline also suggests a CS rate of about 60% in group 8 (19). The practice in BUTH is to allow vaginal delivery in twin pregnancies with the leading twin cephalic, irrespective of parity. This practice is in agreement with the findings of the Twin Birth Study (29). It is noteworthy that in the BUTH series, 75% of the twin deliveries occurred after 33 weeks, and had birth weights greater than 1.7 kg. Also, the babies generally had good 5th minute Apgar scores. Hence, we cannot attribute the relatively low CS rate to pre-viable fetuses or still births. Senior obstetric staff are always available to attend to these deliveries, and are able to safely perform intrauterine manipulations for the delivery of second twin whenever the need arises. This possibly enhanced the incidence and success of planned vaginal twin delivery.

All women in groups 6 (nulliparous breech) and 9 (transverse or oblique lie) had caesarean births. This was not unusual, as these were women who had either foetal malposition or abnormal lie. Similar findings were reported in other studies [14, 23, 24]. It should be noted that the combined relative size of these two groups was just 2.4% of total births, hence, their contribution to the total CS rate was minimal. The CS rates in nulliparous women single cephalic term in spontaneous labour (group 1) and multiparous women without previous CS, single cephalic term in spontaneous labour (group 3) were comparable, being 9.2% and 10% respectively. The major indications for CS in these women were poor progress in labour, cephalopelvic disproportion, and foetal distress in labour. The lower group specific CS rate in group 3 women (16.9%) compared to group 1 women (24.4%) was not unexpected since nulliparous women are more prone to labour dystocia than multiparous women [30].

The commonest indication for CS in this study was previous CS, making up 38% of all indications. This was followed by labour dystocia which accounted for almost 20% of the indications. Previous CS was also the commonest indication in other similar studies, with reported rates of 32% in Lagos, 39% in Tanzania and 35% in Bangladesh [13, 14, 20].

### Implications for CS rate reduction strategies

Consistent use the Robson classification for CS audit will enable the identification of target groups where interventions aimed at reducing CS rates will be most effective [28]. Robson groups 5, 2, 10, and 3 contributed over 70% of the CSs in BUTH during the study period. The findings from this study suggest that efforts directed at reducing the first CS and encouraging VBAC when indicated will have the most significant effect on reducing CS rate. The appropriate use of, and effective protocol for induction of labour when indicated will also help to reduce CS rate. Effective counselling of intending parturients, continuous labour support, pain management including clear agreement on availability of epidural analgesia and high level adherence to evidence-based clinical guidelines are some of the other measures that may encourage women to opt for vaginal delivery and have a satisfactory experience in labour. The effective implementation of these strategies will ultimately lead to safe reduction in CS rate [6].

### Strengths and limitations

This study represents to the best of our knowledge, the first documentation of the analysis of CS using Robson classification in a private teaching health facility in Nigeria. The analysis of the relative contribution of each Robson group to the CS burden is also a strength of this study. This study however has some limitations that should be considered. Some of the stated indications for CS could not be validated since the data was collected retrospectively from case files. Also, considering the fact that the study was done in a single tertiary health facility with a significant burden of referred cases, some of the findings might not be generalizable. This justifies the need for future research on this topic in other large private health facilities in Nigeria.

## Conclusions

The CS rate in BUTH was 51.2%; Robson groups 5, 2, 10, and 3 were the major contributors to this high rate. Interventions directed at reducing the first CS by improving management of spontaneous and induced labours; and strengthening clinical practice around encouraging vaginal birth after caesarean section will have the most significant effect on reducing caesarean section rate in the institution.

## Data Availability

The datasets supporting the conclusions of this article are included within the article.
